# Gas–Liquid Mass Transfer Behavior of Upstream Pumping Mechanical Face Seals

**DOI:** 10.3390/ma15041482

**Published:** 2022-02-16

**Authors:** Shaoxian Bai, Jialin Hao, Jing Yang, Yuansen Song

**Affiliations:** 1 College of Mechanical Engineering, Zhejiang University of Technology, Hangzhou 310032, China; haojialin2019@163.com (J.H.); yangjing@zjut.edu.cn (J.Y.); songyuansen0704@163.com (Y.S.); 2 Institute of Process Equipment and Control Engineering, Zhejiang University of Technology, Hangzhou 310032, China

**Keywords:** gas–liquid mass transfer behavior, mechanical face seals, elliptical grooves, cavitation effect, upstream pumping effect

## Abstract

For gas–liquid medium isolation seals in aero-engines, the upstream pumping function of directional grooves provides an effective way to realize the design of longer service life and lower leakage rate. However, this produces a new problem for gas–liquid mass transfer in the sealing clearance. This study establishes an analytical model to investigate the gas–liquid mass transfer behavior and the change rule for the opening force of mechanical face seals with elliptical grooves. Compared with traditional studies, this model considers not only the gas–liquid transfer but also the cavitation effect. The results obtained show that with the increase of rotational speed, the gas medium transferred from the inner low-pressure side to the outer high-pressure side. In addition, the leakage rate of the liquid medium from the outer high-pressure side to the inner low-pressure side increased with the growth of sealing clearance, rotational speed and seal pressure. The upstream pumping effect of the gas medium with elliptical grooves not only led to a state of gas–liquid mixed lubrication in the sealing surfaces, but also significantly increased the opening capacity of the seal face. This research may provide a reasonable basis for the design of upstream pumping mechanical face seals.

## 1. Introduction

The face seal is a form of sealing widely used in aero-engines to achieve gas–liquid medium isolation in complex oil and gas working environments [1,2,3,4,5]. In order to reduce sealing wear, micro-grooves were proposed to improve sealing surfaces. It was later found that the micro-grooves on the sealing surfaces have an upstream pumping effect, pumping oil or gas from the low-pressure side to the high-pressure side, which can allow for effective separation of the oil and gas. Therefore, this kind of seal may be referred to as an “oil–seal–gas” seal or a “gas–seal–liquid” seal [6].

For upstream pumping liquid seals, the face groove is required to have good liquid upstream pumping characteristics. Numerous studies have been conducted to investigate the upstream pumping effect of seals with spiral grooves [7,8,9,10]. For example, Salant and Homiller [11] used numerical analysis to study the lubricating film in spiral-groove upstream pumping mechanical seals considering cavitation. They found that a properly designed spiral groove may perform well in a double-seal arrangement. Moreover, Lai [12] developed several seals based on spiral groove pumping principles and confirmed that the upstream pumping effect of the spiral groove on the fluid can reversely pumpthe medium from the low-pressure side to the high-pressure side so as to reduce leakage, achieving “zero” leakage of the sealing medium. At the same time, scholars have also carried out numerical studies on the sealing performance of the upstream pumping effect of mechanical seals with spiral grooves, which is influenced by operating parameters such as film thickness and rotational speed, as well as structural parameters such as groove depth and spiral angle [13]. The spiral groove on the surface of mechanical seals showed an obvious hydrodynamic pressure effect, which effectively increases the capacity of the seal opening force.

After that, researchers used both numerical simulations and experimental methods to study mechanical face seals with spiral grooves, T-grooves or inclined elliptical grooves [14,15,16,17,18,19,20,21]. The results showed that good dynamic pressure characteristics and friction characteristics could be obtained under the liquid lubrication condition, indicating that the structure of the inclined elliptical grooves had a better pumping effect than that of the spiral grooves. This may provide new guidance for the groove design of mechanical seal surfaces.

At present, Lebeck’s experimental research based on the upstream pumping seal of a linear tank showed that the gas–seal–oil seal of high-pressure oil could be realized through the design of the face grooves [22]. The feasibility of gas-phase and liquid-phase heterogeneous fluid mutual sealing technology can also be confirmed. However, in contrast to investigations of phase-invariant lubrication of gas seals, the existence of cavitation effects in the groove region [23,24] complicates the upstream pumping law of porous seal faces when a phase change between liquid and gas is taken into account.

Therefore, it is necessary not only to know the bearing capacity of gas–liquid medium but also to determine the law of medium transfer for the seal design. This study aims to establish a theoretical model to analyze the upstream pumping effect of mechanical face seals with inclined elliptical grooves while taking the cavitation effect into account. The gas–liquid mass transfer behavior and the open force change rule are investigated under the different sealing clearances, rotational speeds, sealing pressures and groove parameters. The proposed model has the potential to predict the sealing performance of mechanical face seals. The conclusions drawn based on the simulation results may provide insightful information for engineering seal design and analysis, as well as for the optimal design of surface grooves.

## 2. Theoretical Modeling

### 2.1. Model Description

**Figure 1:**
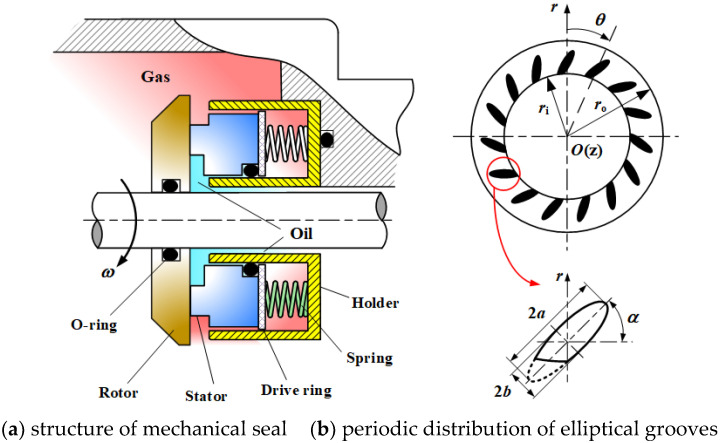
Schematic of the mechanical face seal with inclined elliptical grooves. (**a**) structure of mechanical seal; (**b**) periodic distribution of elliptical grooves.

The structure of mechanical face seals with inclined elliptical grooves is shown in Figure 1. It is clear that the function of this seal is to prevent leakage from the high-pressure oil side to the low-pressure gas side, as shown in Figure 1a. The elliptical grooves are formed on the surface of the rotor ring and are evenly distributed in the radial direction, as shown in Figure 1b. The slender ratio *γ* of the elliptical groove can be defined as the value of its long axis radius *a* versus its short axis radius *b*. *α* is the inclination angle of the elliptical grooves with respect to the radius direction of the rotor ring. *n*_r_ is the number of radial single rows of holes and *n*_θ_ is the number of circumferential rows of holes. *r*_i_ and *r*_0_ are the inner diameter and outer diameter of the rotor ring, respectively.

Based on the practical engineering working conditions of air engines, the geometric parameters and working conditions of the mechanical face seal with elliptical grooves used in this numerical simulation are shown in Table 1.

### 2.2. Control Equation

Naturally, the cavitation effect can be induced by the phase transition of fluid from liquid to gas. Considering the compressibility of the fluid, the Reynolds equation still applies for lubrication analysis in the cavitation region. Due to the periodic distribution of grooves, a radial grooved column was selected as the research object. Therefore, the Reynolds equation with the polar coordinate form could be used in this simulation as follows [25]:

(1) $$\frac{\partial }{r\partial \theta }\left(\frac{\rho h^{3}}{\eta }\frac{\partial p}{r\partial \theta }\right)+\frac{\partial }{r\partial r}\left(\frac{\rho h^{3}}{\eta }\frac{\partial p}{\partial r}\right)=6\omega \frac{\partial \left(\rho h\right)}{\partial \theta }$$

where *ρ* is the density of the fluid, *ω* is the circumferential velocity, *p* is the local pressure, *h* is the local film thickness, *η* is the viscosity of the medium, and *r* and *θ* are the polar coordinates.

### 2.3. Mixed Fluid Flow Considering Cavitation Effect

The film gas–liquid ratio is commonly defined as

(2) $$\lambda_{\mathrm{GL}}=\frac{V_{\mathrm{gas}}\frac{p}{p_{a}}}{V_{\mathrm{gas}}\frac{p}{p_{a}}+V_{\mathrm{liq}}}$$

where *P*_a_ is the standard atmospheric pressure, and *V*_gas_ and *V*_liq_ are the volume of gas and liquid at standard atmospheric pressure, respectively.

Then, the film density can be expressed as follows:

(3) $$\rho =\lambda_{\mathrm{GL}}\rho_{0,\mathrm{gas}}\frac{p}{p_{a}}+(1-\lambda_{\mathrm{GL}})\rho_{\mathrm{liq}}$$

where *ρ*_0,gas_ and *ρ*_liq_ are the gas and liquid densities at standard atmospheric pressure, respectively.

It should be noted that the compressibility of fluid in lubrication theory is mainly represented by the density variation. We assumed that the fluid in the cavitation region existed in a completely gaseous state. Then, the cavitation pressure *p*_c_ can be defined as the critical pressure at which the phase state changes from liquid to gas. At this pressure, the calculated density should be equal to the liquid-state density *ρ*_0,liq_ at standard atmospheric pressure. Therefore, the following state equation should be satisfied in the cavitation region:

(4) $$\frac{\rho_{\mathrm{liq}}}{p}=\frac{\rho_{0,\mathrm{liq}}}{p_{c}^{}}\;\;p\leq p_{c}$$

Therefore, we can use the following density equation to describe the cavitation effect in the liquid lubrication analysis:

(5) $$\{\begin{array}{cc} \rho_{\mathrm{liq}}=\rho_{0,\mathrm{liq}} & \begin{array}{cc} \mathrm{if} & p>p_{c} \end{array}\\ \frac{\rho_{\mathrm{liq}}}{p}=\frac{\rho_{0,\mathrm{liq}}}{p_{c}^{}} & \begin{array}{cc} \mathrm{if} & p\leq p_{c} \end{array} \end{array}$$

Further, the film viscosity can be calculated by

(6) $$\eta =\lambda_{\mathrm{GL}}\eta_{0,\mathrm{gas}}+(1-\lambda_{\mathrm{GL}})\eta_{0,\mathrm{liq}}$$

where *η*_0,gas_ and *η*_0,liq_ are the viscosity of gas and liquid, respectively. Here, the influence of pressure on fluid viscosity was not considered.

### 2.4. Pressure Boundary Conditions

In order to ensure the reliability of the calculation, the accurate calculation of the flow conservation is extremely important, especially for the application of seal design with surface grooves. In fact, the leakage rate, as one of the most important sealing performance, is the flow rate in the radial direction for the face seal. Based on the previous study of our group, the flow conservation of the Reynolds equation can be guaranteed by the finite difference method. The pressure distribution on the periodic calculation region can be obtained by this method. Generally, inlet and outlet pressures are assumed to be constant. Therefore, in the numerical analysis, the following pressure boundary conditions were applied:

(7) $$p(r=r_{i},\theta )=p_{i}$$

(8) $$p(r=r_{o},\theta )=p_{o}$$

(9) p(r,θ=π/N)=p(r,θ=−π/N)

Equations (7) and (8) are the mandatory pressure boundary conditions for the liquid face seal. Since the inclined elliptical grooves are distributed periodically along the circumference direction, we selected a period in the numerical calculation to reduce the computational amount. The periodic pressure boundary condition in this calculation can be described as Equation (9).

### 2.5. Medium Boundary Conditions

Similarly to the pressure boundary condition, the medium boundary conditions can be applied in the numerical analysis:

(10) $$\lambda_{\mathrm{GL}}(r=r_{i},\theta )=1$$

(11) $$\lambda_{\mathrm{GL}}(r=r_{o},\theta )=0$$

(12) $$\lambda_{\mathrm{GL}}(r,\theta =\pi /N)=\lambda_{\mathrm{GL}}(r,\theta =-\pi /N)$$

Equations (10) and (11) are the mandatory medium boundary conditions for the liquid face seal. The periodic medium boundary condition can be described as Equation (12).

### 2.6. Numerical Method

In this model, the formula of the open force is expressed as

(13) $$F_{\mathrm{open}}=\int_{0}^{2\pi }\int_{r_{i}}^{r_{o}}prdrd\theta$$

The gas leakage rate is expressed as

(14) $$Q_{\mathrm{gas}}=-\frac{1}{12\eta }\int_{0}^{2\pi }h^{3}r\lambda \frac{\partial p}{\partial r}d\theta$$

The liquid leakage rate is expressed as

(15) $$Q_{\mathrm{liq}}=\frac{1}{12\eta }\int_{0}^{2\pi }h^{3}r(1-\lambda )\frac{\partial p}{\partial r}d\theta$$

The convergence criterion can be defined as

(16) $$\delta X=\left|\frac{X^{j}-X^{j/2}}{X^{j}}\right|$$

where $X=\left\{p,\lambda_{\mathrm{GL}}\right\}$, and *j* is the iterative number.

**Figure 2:**
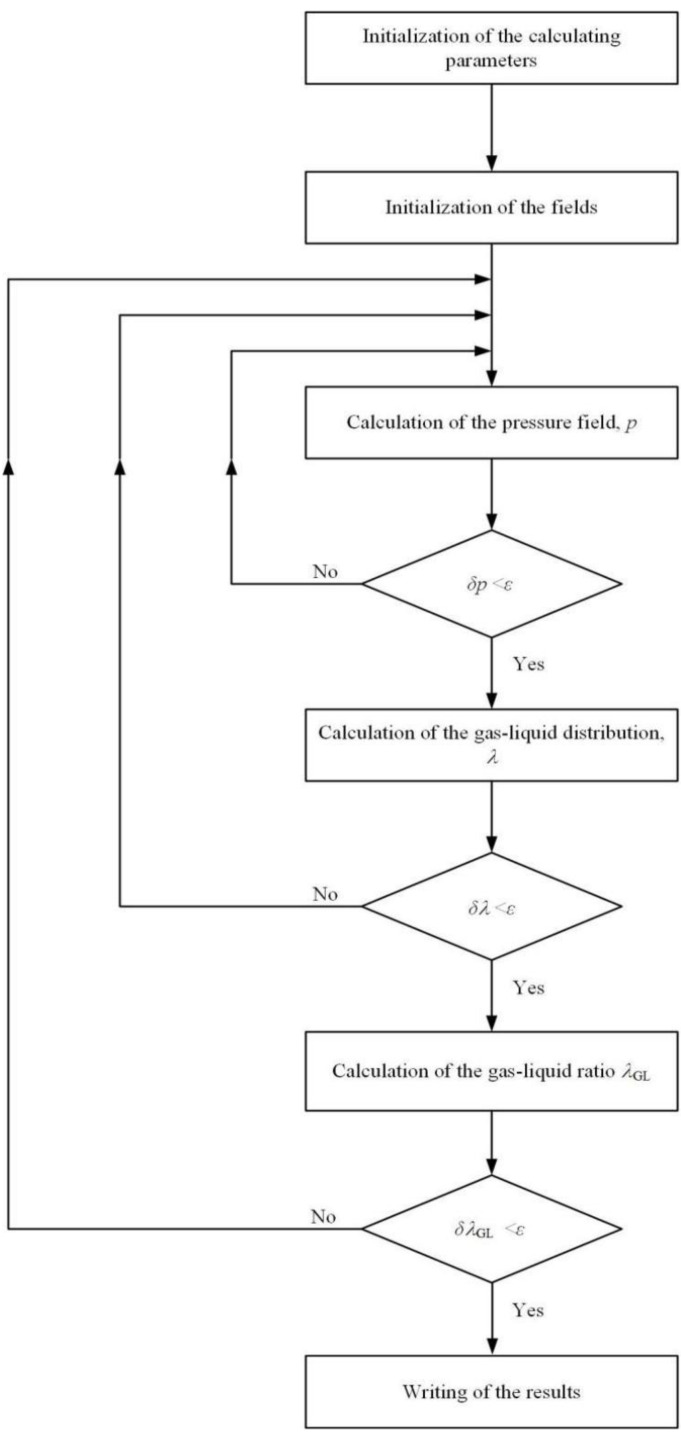
Flowchart of the numerical procedure.

In this study, the finite difference method and appropriate discrete difference scheme were adopted to guarantee the conservation of flow during calculation and the reliability of the simulation. The detailed algorithm of the program is illustrated in Figure 2. Firstly, the initial value of this calculation is inputted, containing the mesh density of the gas film (120 × 80) and the error limit of the convergence criterion (*ε* is fixed as 10^−5^). Then, the film pressure, gas–liquid distribution and gas–liquid ratio are successively calculated into three overlapping loops. The new equilibrium position of the seal clearance is then established, and the entire iterative process is repeated until the convergence criterion on the gas–liquid ratio is satisfied.

### 2.7. Model Validation

In order to validate the model, the leakage rates between the referenced work of Lebeck [22] and this proposed model were compared. In Lebeck’s experimental study, no oil leakage was found. His further theoretical results show that the gas leakage was 0.004 L/min, the oil leakage was 0 and the oil flow in/out at the outside diameter was 0.75 g/h.

**Figure 3:**
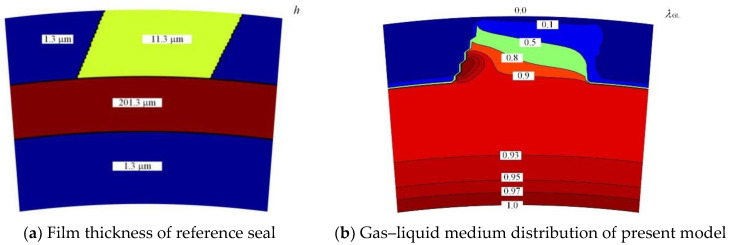
Numerical results of reference seal obtained using present model: (**a**) distribution of film thickness of reference model [22] and (**b**) gas–liquid medium distribution of present model. (*ω* = 7000 rpm, *p*_o_ = 0.101 MPa, *p*_i_ = 0.102 MPa and *h*_0_ = 1.3 μm).

The theoretical results of the present model are shown in Figure 3 according to the simulation parameters from Lebeck’s work. The calculation parameters used in the comparison are also listed in Figure 3a, where 0.101 MPa foamy oil is located at the outside diameter and 0.102 MPa air at the inside diameter. The theoretical gas–liquid medium distribution of the present model is shown in Figure 3b. It can be seen that the value of the gas–liquid ratio of the film was *λ*_GL_ = 1 at the inner diameter, which means that there was only gas leakage from the inner side to the outer side of the seal, but no liquid leakage. The theoretical value of the gas leakage was about 0.003 L/min, and that of the oil leakage was 0. In addition, the value of the film gas–liquid ratio was not equal to 1 and fluctuated near 0, which means that some oil flowed into the seal clearance and then flowed out at the outer diameter. The oil flow in/out at outside diameter was about 0.57 g/h. As a whole, the theoretical results of the present model are in good agreement with Lebeck’s work.

## 3. Results and Discussion

**Figure 4:**
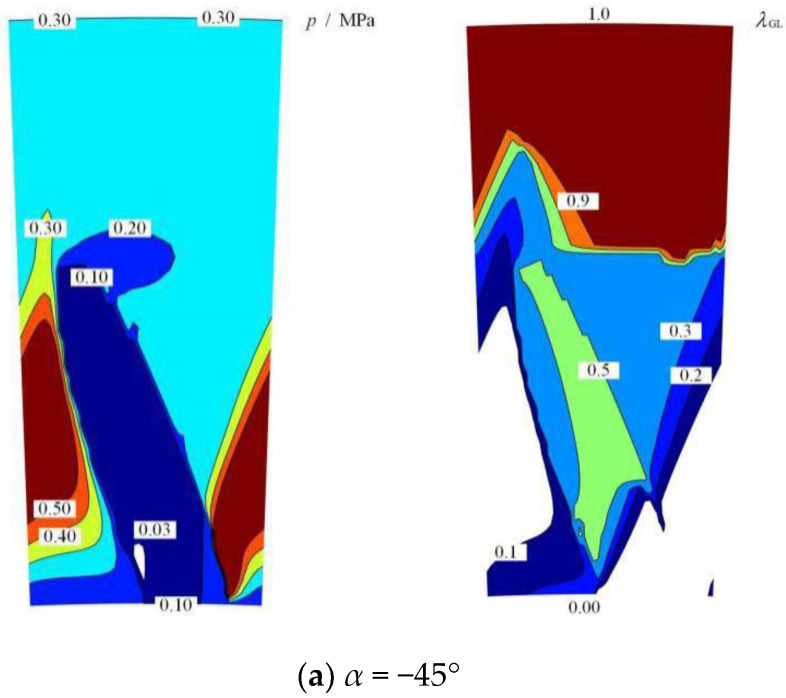

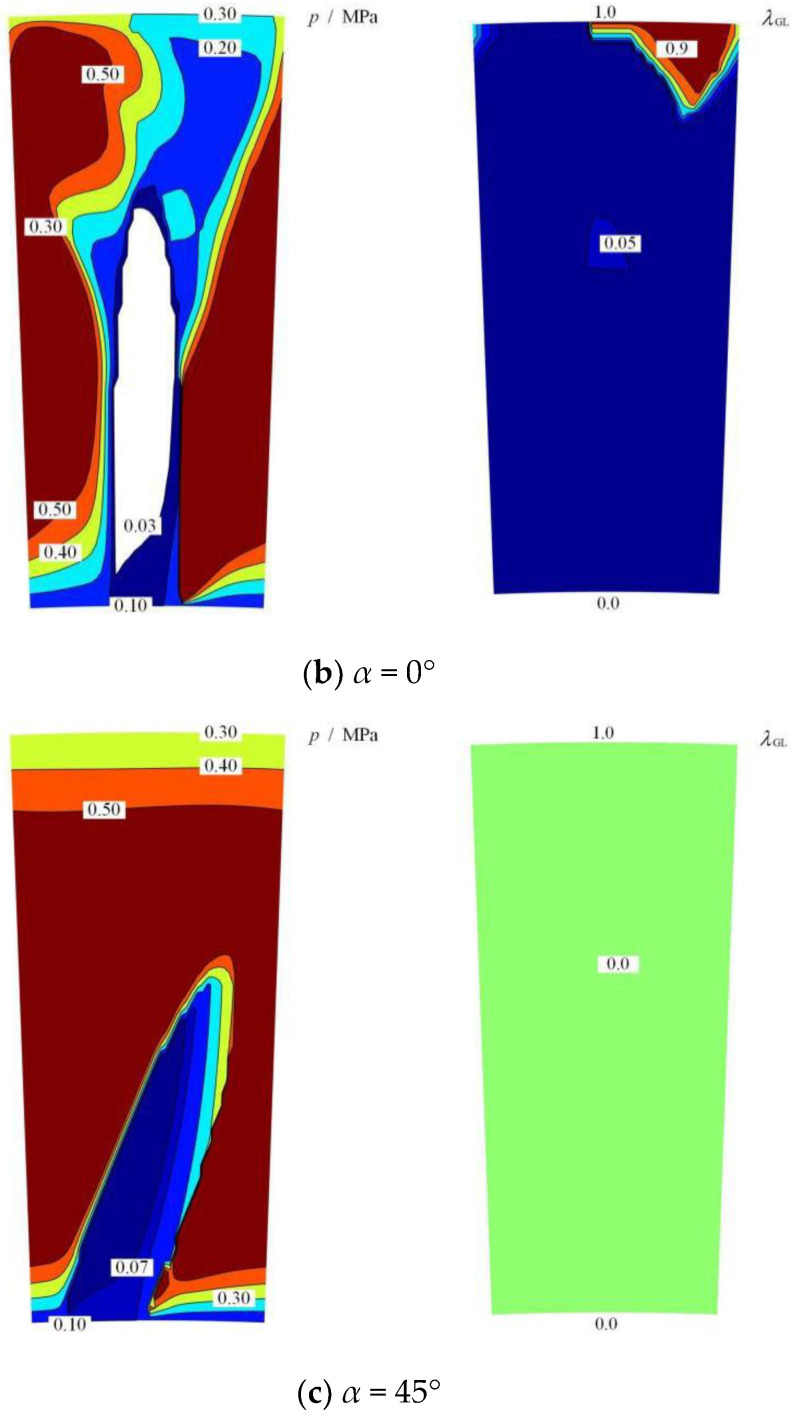
Distributions of film pressure and gas–liquid medium with various inclined angles: (**a**) *α* = −45°, (**b**) *α* = 0° and (**c**) *α* = 45° (*ω* = 2000 rpm, *p*_o_ = 0.3 MPa, *h*_0_ = 2 μm, *γ* = 3.5 and *N* = 36).

In this numerical study, the geometrical parameters and working conditions can be seen in Table 1. Based on these parameters, the distributions of fluid film pressure and gas–liquid medium between the seal surfaces with various inclined angles can be obtained, as shown in Figure 4.

In fact, the sealing face was in a state of gas–liquid mixed lubrication. Not only did the liquid leak from the high-pressure side to low-pressure side driven by the pressure flow, but the gas leaked from the low-pressure side to high-pressure side driven by the shear flow and upstream pumping grooves.

Generally, there is a mass transfer of the sealed medium flowing from the high-pressure side to the low-pressure side driven by the seal pressure, which is called forward leakage. Otherwise, the fluid may be pumped from the low-pressure side to the high-pressure side due to the upstream pumping function of face grooves under the shear effect of the rotational speed, resulting in a backward pumping leakage.

As illustrated in Figure 4, the elliptical grooves on the sealing surface can produce obvious backward pumping effects as well as hydrodynamic pressure effects, where the liquid fluid is set at the low-pressure side with a pressure of 0.1 MPa and the gas fluid is at the high-pressure side with a pressure of 0.3 MPa. As shown in Figure 4a, when the inclined angle *α* = −45°, the shear effect of the rotational speed produced an obvious lower-pressure region of 0.1 MPa in the groove area and a higher-pressure region of 0.5 MPa in the smooth area. The more important fact is that some liquid medium was pumped from the low-pressure side to the high-pressure side along the groove. In the groove area, the gas–liquid ratio decreased to about 0.5, while which reached about 0.9 near the high-pressure side. This means that some liquid medium was pumped into the sealed gas medium, forming backward pumping leakage of the liquid medium. This phenomenon indicates that obvious gas–liquid transfer behavior is produced between sealing clearance of the mechanical seal with elliptical grooves.

Furthermore, when the inclined angle *α* = 0°, as shown in Figure 4b, an obvious cavitation region with pressure 0.03 MPa was formed in the groove area, along with a larger high-pressure region of 0.50 MPa nearly crossing from inner radius to the outer radius. At the same time, the leakage gas–liquid ratio in the seal clearance was lower than 0.05, indicating that the seal was trending toward full backward pumping.

Clearly, Figure 4c presents a complete backward pumping phenomenon when the inclined angle *α* = 45°, where the value of the leakage gas–liquid ratio in the seal clearance was equal to 0, and only the low-pressure liquid was transferred to the high-pressure side without high-pressure gas leaking. This means that the sealing performance under the simulated conditions can avoid the sealed gas medium leakage.

In the following analysis, we analyze the influence of operating parameters and groove parameters on the gas–liquid mass transfer behavior.

### 3.1. Operating Parameters

#### 3.1.1. Seal Clearance

**Figure 5:**
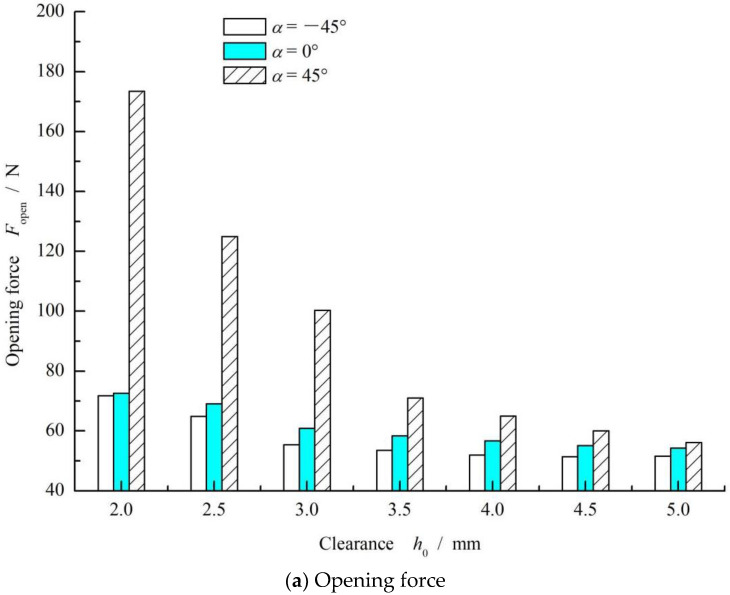

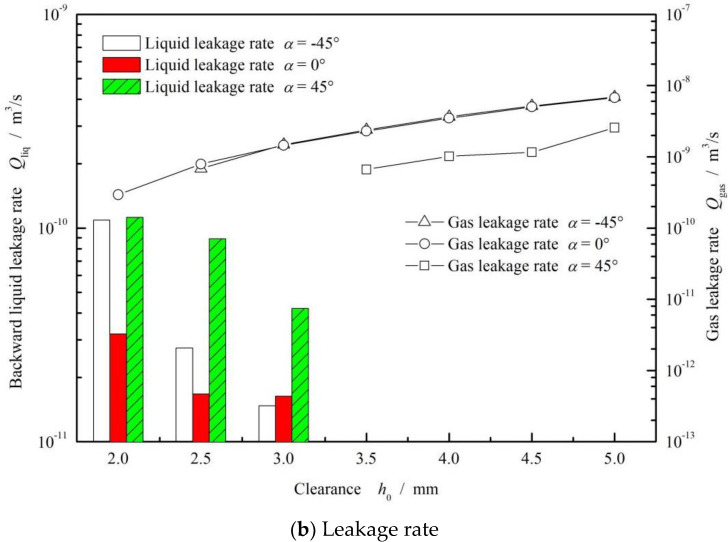
Effect of seal clearance on the opening force (**a**) and leakage rate (**b**) (*ω* = 10,000 rpm, *p*_o_ = 0.3 MPa, *h*_0_ = 2 μm, *γ* = 3.5 and *N* = 36).

Figure 5 shows the influence curve of seal clearances on the opening force and leakage rate. As can be seen from Figure 5a, for the case of *α* = 0° and *α* = 45°, the opening force curve shows a significantly decreasing tendency with an increase in sealing clearance. For the cases in which *α* = −45°, the value of the opening force increased gradually with increasing clearance. In addition, the inclined angle had a significant influence on the opening force. The maximum opening force value at *α* = 45° was about three times larger than that at *α* = −45°.

For *α* = 0° and *α* = −45°, there was no backward liquid leakage rate when the clearance was larger than 2.5 μm. While in the case of *α* = 45°, there was an increasing trend in the backward liquid leakage rate with increasing clearance. In addition, the forward gas leakage rate increases with increasing clearance. The reason for this result may be that, with the increase in clearance, the shear rate of the fluid decreases, which leads to a decreasing of the upstream pumping effect, such that the gas leakage increases and the liquid leakage rate decreases. However, the result of the inclined angle *α* = 45° had a more significant upstream pumping effect than that of *α* = −45° and *α* = 0°, which led to a larger backward liquid leakage rate and a larger hydrodynamic opening force, as discussed in [26,27].

Another important result is that, for the case of *α* = 45°, when the clearance was smaller than 3 μm, there was only backward liquid leakage rate and no gas leakage occurred. This means that high speed may form zero leakage of sealed gas for the inclined elliptical groove face seals.

#### 3.1.2. Rotational Speed

**Figure 6:**
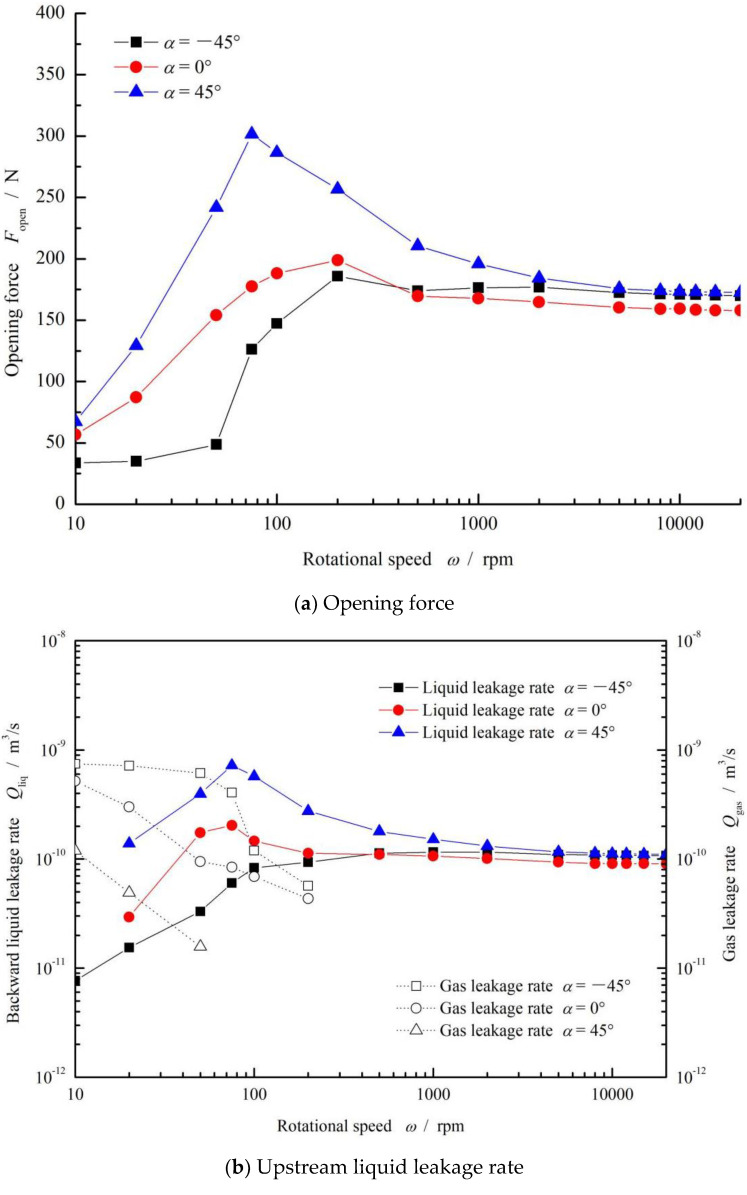
Effect of rotational speed on the opening force (**a**) and leakage rate (**b**). (*p*_o_ = 0.3 MPa, *h*_0_ = 2 μm, *γ* = 3.5 and *N* = 36).

The influence of rotational speed on the opening force, gas leakage rate and gas–liquid ratio is studied in this section, as shown in Figure 6. According to Figure 6a, it can be found that the opening force demonstrated a tendency of first increasing and then decreasing with increases in rotational speed from 0 to 100 rpm. The maximum value of the opening force appeared when the speed reached a range of 100 to 300 rpm. After this, as the speed increased, the opening force gradually decreased to the lowest value and then slowly increased. Finally, the increase in the opening force exceeded 100% compared to the initial value when the speed was equal to zero.

The change rule of the opening force with increasing speed may be due to the upstream pumping effect caused by the grooves on the sealing surfaces. Specifically, the fluid flows into the groove areas under the speed shearing effect. On the one hand, due to the existence of groove depth, the flow resistance decreases, so that a part of the fluid flows along the long axis direction of the grooves and then produces an upstream pumping effect from the low-pressure side to the high-pressure side. At the same time, the extrusion action of the fluid may produce a dynamic pressure effect and increase the opening force. On the other hand, since the flow space is divergent, the pressure drops suddenly and reaches the cavitation pressure, forming a cavitation region. This may impede the flow of fluid along the radial direction, thereby weakening the upstream pumping effect. As the speed increases, the cavitation effect becomes prominent and the upstream pumping influence increases, resulting in the inflection point of the opening force at a speed of around 100~300 rpm.

For the fluid mass transfer, according to Figure 6b, the gas leakage rate and gas–liquid ratio show an obvious decreasing tendency as a whole with increasing rotational speed. When the speed was greater than 300 rpm, there was no gas leakage. This means that the mass transfer of the low-pressure liquid to the high-pressure side was enhanced due to the shear effect, as discussed in the section above. Correspondingly, the mass transfer of high-pressure gas to the low-pressure side was restrained.

As discussed in the section above, we can conclude that high speeds may result in zero leakage of sealed gas for inclined elliptical groove face seals.

#### 3.1.3. Seal Pressure

**Figure 7:**
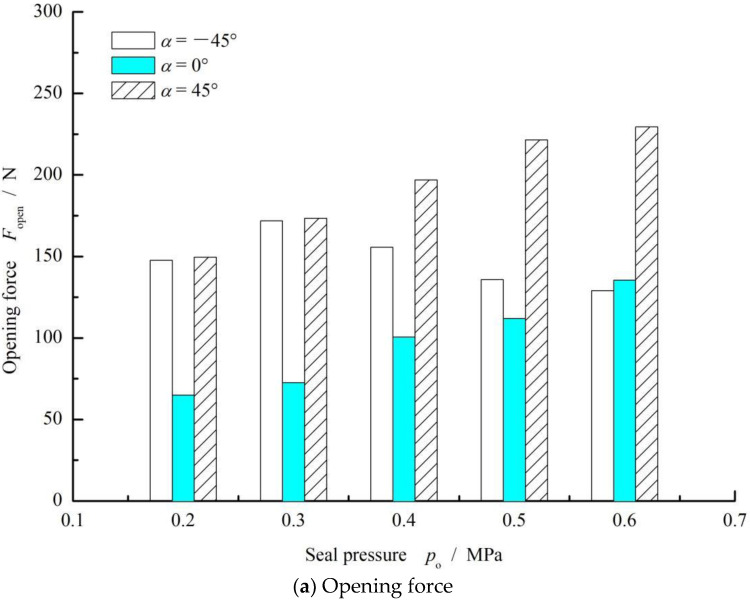

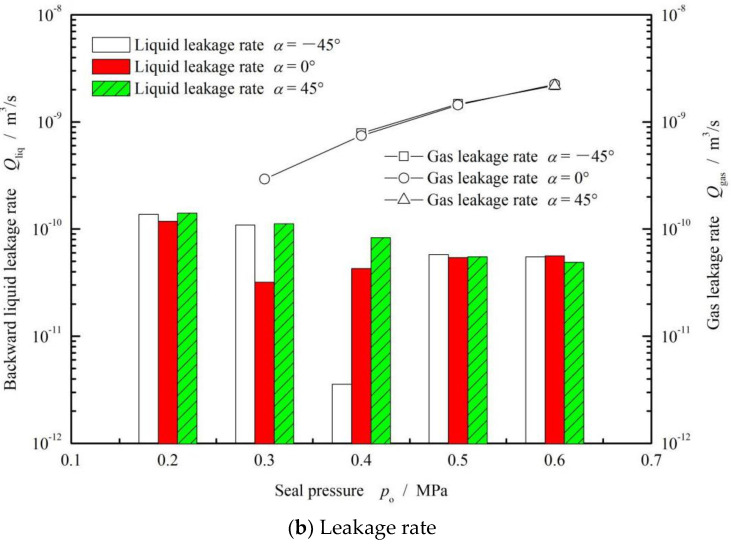
Effect of seal pressure on the opening force (**a**) and leakage rate (**b**). (*ω* = 10,000 rpm, *p*_o_ = 0.3 MPa, *h*_0_ = 2 μm, *γ* = 3.5 and *N* = 36).

Figure 7 shows the influence of seal pressure on the opening force and leakage rate. As can be seen from this figure, with the increase in sealing pressure, the change rules for these three performances all show a stable increasing tendency. The main reason for this change is that the opening force consists of two parts: one part comes from the pressure distribution formed by seal pressure mainly in the smooth region, whereas the other part results from the pressure distribution formed by shear flow mainly in the grooved region. With the increase of seal pressure, the seal-pressure effect increased while the shear-flow effect decreased. The combination of these two factors caused a stable growth of the opening force.

In addition, with an increase in seal pressure, the gas leakage rate increased quickly while the backward liquid leakage rate decreased. However, for the case of *α* = 45°, there was no gas leakage until the seal pressure increased to about 0.6 MPa due to the larger upstream pumping action of the elliptical grooves.

### 3.2. Groove Parameters

#### 3.2.1. Slender Ratio

**Figure 8:**
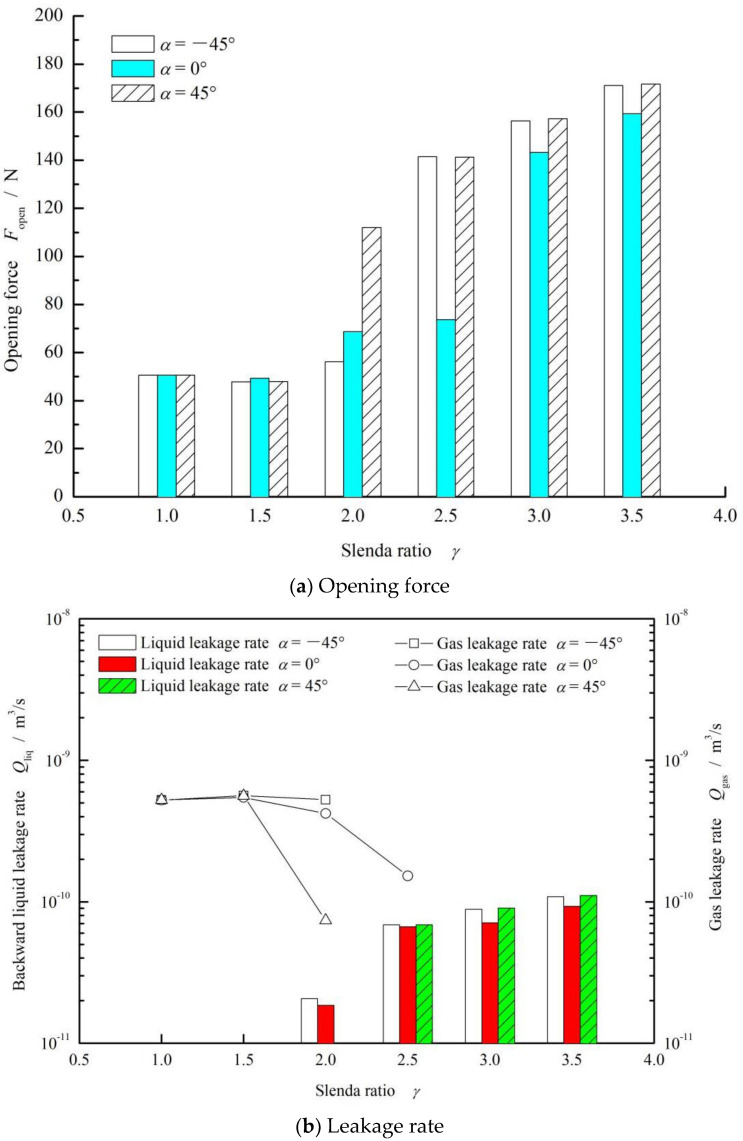
Effect of slenderness ratio of elliptical grooves on the opening force (**a**) and leakage rate (**b**). (*ω* = 10,000 rpm, *p*_o_ = 0.3 MPa, *h*_0_ = 2 μm and *N* = 36).

In the design of face elliptical groove, the strength of the backward pumping effect largely depends on its slender ratio. Figure 8 shows the influence curve of the slenderness ratio of elliptical grooves on the opening force and leakage rate. According to Figure 8a, when the slender ratio was less than 3.5, the opening force tended to increase.

Moreover, the gas leakage showed an obviously declining trend with the growth of the slenderness ratio from 1 to 3.5 in all three cases (*α* = −45°, 0° and 45°). While the slenderness ratio was greater than 2, the gas leakage disappeared and the backward liquid leakage rate increased quickly with the increasing slenderness ratio.

#### 3.2.2. Number of Grooves

**Figure 9:**
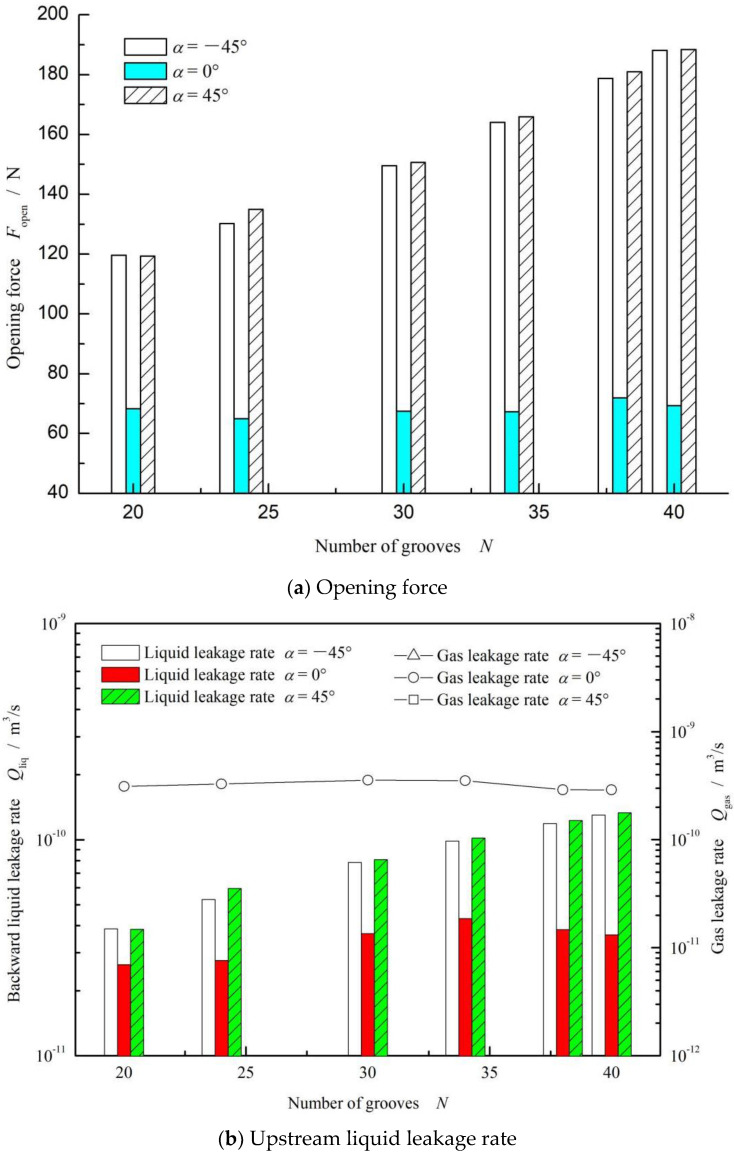
Effect of number of grooves on the opening force (**a**) and leakage rate (**b**). (*ω* = 10,000 rpm, *p*_o_ = 0.3 MPa, *h*_0_ = 2 μm and *γ* = 3.5).

The number of grooves is another important parameter affecting fluid flow and hydrodynamic effects in the clearance. Generally, the larger the number of grooves, the greater the pumping effect. The influence of the number of grooves on the opening force and leakage rate is investigated in this part, as shown in Figure 9. According to Figure 9a, for all three cases, it is clear that the opening force increased as the number of grooves grew.

For the fluid mass transfer shown in Figure 9b, consistent with theoretical expectations, the backward pumping effect was enhanced by increasing the number of grooves, resulting in a phenomenon in which the gas medium transfer is inhibited while the liquid transfer is enhanced. When the number of grooves was increased from 20 to 40, the liquid leakage rate from the low-pressure side to the high-pressure side increased, while the gas leakage rate increased gradually in the case of *α* = 0°. For the cases of *α* = −45° and 45°, there was no leakage of the sealed gas.

## 4. Conclusions

In this paper, an analytical model to investigate the gas–liquid mass transfer behavior and the change rule for the opening force of mechanical face seals with elliptical grooves was established. Compared with the traditional single fluid medium model, the current model provides a method for analyzing the two-way leakage and mutual sealing problems of two different fluid media. The main conclusions can be summarized as follows:

1. The backward pumping effect of liquid medium by elliptical grooves not only led to a state of gas–liquid mixed lubrication on the sealing surfaces, but also significantly increased the opening force and the gas medium transfer from the inner low-pressure side to the outer high-pressure side with increasing rotational speed.
2. The leakage rate of the high-pressure liquid medium from outer high-pressure side to the inner low-pressure side increased with increasing clearance, rotational speed and seal pressure. In addition, the backward pumping effect of the inclined elliptical grooves inhibited the leakage of the high-pressure gas. Zero leakage seals may also be achieved through inclined elliptical grooves.
3. The proposed model can predict the sealing performance of mechanical face seals with elliptical grooves. Through the surface grooves design and optimization, the sealing system can satisfy more demanding working conditions and provide safer operation equipment.

## Figures and Tables

**Table 1:** Parameters of the mechanical face seal.

Item	Symbol	Dimensions and Data	Item	Symbol	Dimensions and Data
Inner diameter	*d*_i_	42 mm	Groove depth	*h*_d_	5 μm
Outer diameter	*d*_o_	49 mm	The short radius of elliptical groove	*b*	0.50 mm
Pressure at inner radius	*p*_i_	0.10 MPa	The slender ratio	*γ*	1~3.5
Pressure at outer radius	*p*_o_	0.2~0.6 MPa	Inclined angle	*α*	−45°~45°
Oil viscosity	*η*	4.9 mPa.s	Groove number along circumferential direction	*N*	20~40
Rotational speed	ω	0~20,000 rpm	Clearance	*h*_0_	2~5 μm

## Data Availability

All data is contained within the article.
